# Information Theoretic Modeling of High Precision Disparity Data for Lossy Compression and Object Segmentation

**DOI:** 10.3390/e21111113

**Published:** 2019-11-13

**Authors:** Ioan Tăbuş, Emre Can Kaya

**Affiliations:** Computing Sciences Unit, Tampere University, FI-33014 Tampere, Finland; emre.kaya@tuni.fi

**Keywords:** lossy disparity map compression, image segmentation, mining the geometry of scenes

## Abstract

In this paper, we study the geometry data associated with disparity map or depth map images in order to extract easy to compress polynomial surface models at different bitrates, proposing an efficient mining strategy for geometry information. The segmentation, or partition of the image pixels, is viewed as a model structure selection problem, where the decisions are based on the implementable codelength of the model, akin to minimum description length for lossy representations. The intended usage of the extracted disparity map is to provide to the decoder the geometry information at a very small fraction from what is required for a lossless compressed version, and secondly, to convey to the decoder a segmentation describing the contours of the objects from the scene. We propose first an algorithm for constructing a hierarchical segmentation based on the persistency of the contours of regions in an iterative re-estimation algorithm. Then, we propose a second algorithm for constructing a new sequence of segmentations, by selecting the order in which the persistent contours are included in the model, driven by decisions based on the descriptive codelength. We consider real disparity datasets which have the geometry information at a high precision, in floating point format, but for which encoding of the raw information, in about 32 bits per pixels, is too expensive, and we then demonstrate good approximations preserving the object structure of the scene, achieved for rates below 0.2 bits per pixels.

## 1. Introduction

### 1.1. Motivation

One of the most important types of information handled in modern imaging applications is the geometry of the scene and of the objects present in the scene. The depth maps convey the geometry of the scene and are needed as such as a separate data, to be explicitly encoded and transmitted in some application areas like industrial computer vision or robotics. In other applications, depth data might not be needed explicitly by the user, but it is still used as an intermediate variable helping in removing the redundancy in stereo and multiview image encoding. The disparity map for a stereo pair is proportional to the reciprocal depth map of the scene for an ideal fronto-parallel stereo optical system [1], and modeling the geometry based on depth or on disparity information satisfies the same goal of extracting relevant geometry information, hence, the algorithms that we present can be applied to both types of data. We consider here two aspects that are usually considered separately: the first is the compression of the geometry data, and the second is the mining of the geometry data for finding the relevant objects and parts of objects.

From the compression perspective, we consider in this paper high precision disparity data occurring in immersive media, which is under standardization in JPEG and MPEG working groups [2,3]. In the following, we give examples where this task is relevant. One example is the standardization of point cloud compression (PCC) with voxelized point clouds having very high precision, from 10 to 30 bits per coordinate. In one encoding methodology, using 2D projections of the point clouds to several planes, one gets high precision depth images that need to be encoded [3]. Another example in the light field compression standardized by JPEG Pleno Light Field [2] is that one needs to extract a simplified model of the disparity map from the known disparity map which might be available in floating point precision for the synthetic imagery. The cost of lossless encoding of the disparity map at the full precision may be justified only at very high overall bitrates. For lower bitrates, one needs to reduce the cost of disparity map data, either using a standard lossy encoder of the initial disparity map data, or by using a model based encoding of the disparity information, as we propose in this paper. For the encoding of synthetic images, which occurs in the gaming industry, the image content is generated using models from which the depth information is available at a high precision for all the points in the scene.

### 1.2. Related Work

Our paper considers two aspects that are extremely well studied separately, but which are seldom tackled together. The first one is a model-based disparity map compression, investigated in the image compression literature, and the second is depth image segmentation, which is investigated thoroughly in the pattern recognition literature, but usually without considering the performance of the implementable algorithms for compressing the segmentation. Related papers can be found in the literature dealing with information theory-based segmentation or with model-based depth map compression.

#### 1.2.1. Methods for Disparity Map Compression

We consider in this paper the compression of high precision depth or disparity data, represented in floating point format or high precision integer format. There is work on the lossless compression of such data [4] but most depth compression papers were concerned with the early datasets of 8 bits per pixel raw formats, or on datasets where depth did not have enough precision to justify polynomial approximations beyond planar models. With high precision depth, one can get significantly better approximations by quadratic surface models, as we do here in this paper, where all polynomial models are quadratic surfaces; we show that very precise detection of the contours of the objects is obtained.

While there is a very rich literature on using polynomial models in the image compression standardization related literature, many of them deal with block-based processing of the image, where the segmentations are represented by using quadtree partitions and, hence, the boundaries of objects are not followed at pixel level. We are interested in region based compression, where the arbitrary-shaped contours in the segmentation are encoded explicitly. We use for arbitrary shape region coding the method dubbed crack-edge-region-value (CERV) in [5], which is a context based method for encoding the contours of arbitrary regions, given in the form of crack-edges (elementary contour element between any two neighbor pixels). In [5], the constant disparity values inside each region are also encoded by a context-based algorithm, resulting in an overall lossless compression of the disparity image. A lossy method for disparity maps using arbitrary-shaped regions and planar models inside each region was proposed in [6], with a further refinement in [7] to include a selection of the best model between a planar model and a constant model, inside each region. In [8], the planar models and the underlying segmentation are obtained through Markov random field modeling, by optimizing an energy function, where the number of planar models is an important parameter, and where examples are shown for up to 50 planar models. A wavelet transform approach to depth modeling and coding is presented in [9], where the contours of the regions and the planar variations in each region are modeled using a flexible model.

The most recent JPEG activity for disparity map compression deals with the compression of breakpoints for improving the compressibility of images having high discontinuities, along the lines of the breakpoint adaptive discrete wavelet transform [10], which was illustrated in [11] for the compression of light field images.

#### 1.2.2. Methods for Image Segmentation and Edge Detection

Edge (or contour) detection has a long history [12,13] and is an essential part in modern computer vision systems. Edges provide useful structural information about a color or depth image. Recent works on edge detection [14,15,16] demonstrate that the field of research underwent a rapid development during the last decade.

A classical and perhaps the most well-known method for edge detection is the Canny Edge Detector [13] which applies numerical optimization relying on a general mathematical formulation of detection and localization criteria. Recent approaches are based on machine learning models. Dollar and Zitnick [14] train structured random decision forests for edge detection. DeepContour [17] utilizes a basic CNN architecture and learns to classify input image patches into different contour shape classes. Xie and Tu [15] formulate edge detection as an image-to-image prediction problem and solve it using fully convolutional neural networks in their “Holistically-Nested Edge Detection” framework. More recently, Liu et al. [16] designed a multiscale network architecture to generate rich hierarchical representations.

Superpixels are perceptually meaningful regions in an image. In several computer vision tasks, superpixels are found to be useful, e.g., for object recognition [18,19], scene labeling [20] and object localization [21]. Selective Search [18] starts with an over-segmentation of an input image generated by the graph-based segmentation in [22]. According to certain similarity measures defined over color, shape and texture of regions, neighbors are iteratively merged into larger regions. A state of the art method for objects proposal is multiscale combinatorial grouping (MCG) [19], which performs grouping of multiscale regions and relies on a fast normalized cut algorithm, and was shown to perform very well on the benchmarks for color image segmentation.

A superpixel has to be confined within a single object, therefore, superpixel segmentation has to be loyal to object boundaries. Unlike semantic segmentation, which aims to segment only the objects from a predefined set of classes (leaving some parts of the image unlabeled), superpixel segmentation aims to include every single pixel in one of the segments. This coincides well with our goals because we aim to interpret and compress the entire image. Moreover, by combining the individual superpixels into larger superpixels, it is possible to obtain a segmentation hierarchy.

Superpixel segmentation and edge detection are related in the sense that they both attempt to capture the edge information in an image. On the other hand, it should be noted that edge detection does not necessarily lead to closed contours and, therefore, it is not always easy to recover regions from an edge map. Arbelaez et al. [23] provide a unified approach to these two problems, which we use in the experimental section.

#### 1.2.3. Methods Combining Image Compression and Image Segmentation

Image compression provides the most efficient description of an image, and therefore, it is the essential tool for making inference based on description length. The minimum description length (MDL) for image segmentation was used in [24], where an image partitioning problem is presented in terms of finding the minimum description of an image according to a descriptive language. The connections between the MDL approach, the region growing and the snakes was studied in [25]. Other image segmentation under MDL approach include: [26], where the regions are constrained to be connected components; [27] proposing learning of Gaussian Mixture Models using description codelength criteria; and [28] where a recursive MDL criterion is used in the framework of graph-cuts, and where similar structures in the images are represented by already described similar structures. All the above papers dealt with segmentation of color images, not of disparity map images, but used the same principle of obtaining a segmentation by minimizing the description length of a certain model, which is in final terms expressed as the codelength of a certain program for reconstructing the image.

For applications on more complex data, the MDL-based segmentation of depth maps was studied in [29] for the lossless compression of light fields.

The MDL principle applied to lossy compression in the form of minimizing the description length for a given distortion can be thought of as another facet of the Kolmogorov structure function [30,31].

Another information theoretic approach for model selection is intersection of confidence intervals ICI. Linear Polynomial Approximations combined with ICI(LPA-ICI). Ref. [32] are state-of-the-art for processing images with noise, being extremely efficient especially with impulsive or speckle noise. In the case of our high resolution disparity images, this noise component is not present, but certainly the use of LPA-ICI and similar techniques are worth pursuing in future research. As some interesting related references, segmentation of ultrasonic images was investigated using ICI selection in [33] and by MDL selection in [34].

### 1.3. Contribution

**Figure 1:**
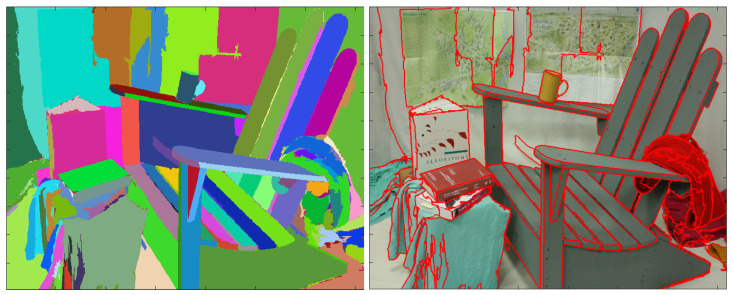
The goal of the paper is to encode disparity images using piece-wise polynomial surface models over the regions of the segmentation. *Left:* one segmentation image obtained using a disparity map; *Right:* The boundaries of segmentation’s regions are overdrawn on the RGB image, showing a very precise match.

This paper has a dual goal: to provide good overall segmentations of the scene, and to achieve efficient lossy compression at low bitrates. Figure 1 shows the main contribution of the paper: obtaining segmentations with very precise contours from disparity images, and using them for model-based lossy compression of disparity maps.

Instead of directly transmitting the raw information, we propose to extract polynomial surface models for the regions belonging to suitable selected partitions of the disparity image. The most challenging task is to obtain a good segmentation of the disparity image, which is achieved from a competition between several possible segmentations. We propose an algorithm belonging to the parametric approach for lossy disparity coding, in which the cost of the models involved will be evaluated in bits, and the task is to seek for the best compromise between the precision of the model and its cost, which has different solutions at different target bitrates.

An important goal for the developed algorithm is to obtain such models of the depth image data that convey information about the objects in the scene. Fitting the polynomial models to the depth data will provide segmentations that capture the most important contours in the image, and will also be able to delineate the objects of interest. We compare the boundaries of the regions in the segmentations that we obtain with our algorithm to the edges obtained from the color images representing the same scene, and we find a high degree of correspondence, which we quantify using the established benchmarking techniques from the segmentation literature.

The main contributions are two segmentation algorithms which produce partitions having simple regions, easy to be transmitted at different desired bitrates to the decoder. During the stage of creating the regions, several models compete for the most efficient image splitting into regions, while ensures a good approximation quality in a procedure based on information theoretic model selection decisions. We analyze the obtained algorithms from the perspective of lossy image compression and from the precision-recall properties of the obtained segmentations.

## 2. Proposed Methods

### 2.1. Definitions and Statement of the Problem

#### 2.1.1. Image Partition into Regions

We introduce first notations and definitions for presenting in a formal way the proposed algorithms. We consider depth or disparities images $G\in R^{n_{r}\times n_{c}}$ having $n_{r}$ rows and $n_{c}$ columns. The set of pixels of the image is denoted $\Omega^{0}=\left\{1,\ldots ,n_{r}\right\}\times \left\{1,\ldots ,n_{c}\right\}$. Two pixels (i,j) and $\left(i^{\prime },j^{\prime }\right)$ are connected in connectivity 4 if $\left(i,j\right)-\left(i^{\prime },j^{\prime }\right)\in \left\{\left(-1,0\right),\left(1,0\right),\left(0,-1\right),\left(0,1\right)\right\}$ and in connectivity 8 if $\left(i,j\right)-\left(i^{\prime },j^{\prime }\right)\in {\left\{-1,0,1\right\}}^{2}$ (unless otherwise specified we assume in the paper that connectivity level is 4). A label image, $X\in N^{n_{r}\times n_{c}}$, specifies a label X(i,j) for each pixel $\left(i,j\right)\in \Omega^{0}$. A region $\Omega_{\ell }\subset \Omega^{0}$ is a subset of $\Omega^{0}$ and is specified in the label image X by the equivalence $\left(i,j\right)\in \Omega_{\ell }\Leftrightarrow X\left(i,j\right)=\ell$. The region is said to be a connected component if for any pixel $\left(i,j\right)\in \Omega_{\ell }$ there is at least one pixel $\left(i^{\prime },j^{\prime }\right)\in \Omega_{\ell }$, which is connected in connectivity 4 to the pixel (i,j). A partition of the image into regions is denoted as a set as $P=\{\Omega_{1},\ldots ,\Omega_{L}\}$ and can be unequivocally described by a label image with *L* labels, which we denote X(P).

#### 2.1.2. Representing the Region’s Contours

The contour, or boundary, of any region is formed of horizontal and vertical crack edges: a horizontal crack edge image $H_{X}\in {\left\{0,1\right\}}^{n_{r}\times n_{c}}$ specifies by $H_{X}\left(i,j\right)=1$ that X(i−1,j)≠X(i,j) and similarly the vertical crack edge image $V_{X}\in {\left\{0,1\right\}}^{n_{r}\times n_{c}}$ specifies by $V_{X}\left(i,j\right)=1$ that X(i,j−1)≠X(i,j) and we define the contour matrix for the label matrix X to be the concatenated matrix $C_{X}=\left[\begin{array}{cc} H_{X} & V_{X} \end{array}\right]$. A crack edge is also named contour element. A label image X has associated a unique contour matrix $C_{X}=\left[\begin{array}{cc} H_{X} & V_{X} \end{array}\right]$. Conversely, a contour image C can be processed by a region labeling routine, X=RegLab(C), to construct a label image X where each connected component has a distinct label, (see, e.g., the BSD benchmarking software [35]). We note that for any label matrix X where each region is a connected component, it holds that $X^{\prime }=\mathrm{RegLab}\left(C_{X}\right)$ differs from X only by a permutation of the labels. The set of contour elements set to one in $C_{X}$ that form the outside border of a region Ω is denoted Γ(Ω).

#### 2.1.3. Representing a Hierarchical Segmentation

In the literature dealing with hierarchical segmentations, the representation of a sequence of segmentations is given by the ultrametric contour map (UCM) which can be formally defined as a contour matrix $U\in R^{n_{r}\times 2n_{c}}$ having real entries, as opposed to the contour matrix C, which has binary elements. It is usual to normalize the real value of the contour element U(i,j) to the range [0;1] and then consider the value as the probability that a contour element separates two adjacent pixels having different labels. However we keep the UCM matrix to be integer-valued, with the elements specifying a persistency level. By thresholding the elements of the UCM matrix U at a threshold $\tau_{\ell }$ one obtains a binary matrix $C_{\ell }$. Using a decreasing sequence of thresholds one obtains a sequence of binary contour images $C_{1},\ldots ,C_{L}$, corresponding to nested segmentations $X_{1},\ldots ,X_{L}$ of the image G, which together form a hierarchical segmentation.

Considering two consecutive nested segmentations $X_{\ell }$ and $X_{\ell +1}$, and two neighbor regions, $\Omega_{\ell_{1}}$ and $\Omega_{\ell_{2}}$, in $X_{\ell +1}$ that were obtained by splitting a single region $\Omega_{\ell_{1,2}}$ in $X_{\ell }$. The split is obtained by setting to one the contour elements from the set $\Delta \Gamma =\Gamma \left(\Omega_{\ell_{1}}\right)\cap \Gamma \left(\Omega_{\ell_{2}}\right)$. The cost of the split in terms of bitrate, L(ΔΓ), can be approximated to be proportional to the number of contour elements in the set ΔΓ, hence L(ΔΓ)=c|ΔΓ|, as is done in most papers using MDL merging-splitting optimization [7,24].

#### 2.1.4. Polynomial Surface for Approximating the Disparity Map over a Region

We consider the following two dimensional polynomials: $P_{0}\left(i,j\right)=\theta_{0}$, $P_{1}\left(i,j\right)=\theta_{0}+\theta_{1}i+\theta_{2}j$, and

(1) $$\begin{array}{c} P_{2}\left(i,j\right)=\theta_{0}+\theta_{1}i+\theta_{2}j+\theta_{3}ij+\theta_{4}i^{2}+\theta_{5}j^{2} \end{array}$$

and denote generically $P_{\theta }\left(i,j\right)=\varphi_{k}{\left(i,j\right)}^{T}\theta$ where the elements of the regression vector $\varphi_{k}\left(i,j\right)$ are monomials in the variables *i* and *j*. The main model considered in this paper is the reconstruction $S\left(\Theta ,P\right)\in R^{n_{r}\times n_{c}}$ of the image G, as a function of a partition $P=\{\Omega_{1},\ldots ,\Omega_{L}\}$ and a set of polynomial parameter vectors $\Theta =\{\theta_{1},\ldots ,\theta_{L}\}$, where the reconstruction surface S for a pixel (i,j) belonging to region $\Omega_{\ell }$ is obtained with the parameters $\theta_{\ell }$, as $S_{i,j}=P_{\theta_{\ell }}\left(i,j\right)$.

Finally, we denote the code length necessary for representing the parameters as $L\left(\Theta \right)=\sum_{\ell =1}^{L}L\left(\theta_{\ell }\right)$, where we assume that the elements of $\theta_{\ell }$ are quantized to a finite precision and are encoded by Golomb–Rice coding (hence assuming a geometric distribution of the parameters). The image of contours $C_{X}$ is encoded by the CERV algorithm [5] and the resulting codelength is denoted $L_{C}\left(C_{X}\right)$, or for short L(X).

The goal of this paper is to start from a given disparity map image G and to find a sequence of partitions $P_{1},\ldots ,P_{N}$ (or equivalently a sequence of label images $X_{1},\ldots X_{N}$) and the corresponding polynomial models $\Theta_{1},\ldots ,\Theta_{N}$ satisfying two desiderata:

1. the rate-distortion description $\left(R_{n},D_{n}\right)$, with $R_{n}=L_{C}\left(C_{n}\right)+L\left(\Theta_{n}\right)$ and $D_{n}={∥G-S\left(\Theta_{n}\right)∥}^{2}$, should be competitive with the rate-distortion of lossy compression algorithms, at very low bitrates. The wish is to extract relevant information from G, to encode it efficiently, and use it for obtaining a reconstruction with a small distortion, as in the lossy compression tasks, but with the next additional wish on the relevance of the segmentation for the objects in the image.
2. The sequence of partitions $P_{1},\ldots ,P_{N}$ should compare favorably with the hierarchical partitions obtained from the color information of the same scene, having the diagram (recall, precision) competitive with the existing state of the art boundary detection or segmentation algorithms for finding general structure in images.

#### 2.1.5. Statement of the Problem

We start by defining the disparity map model, consisting of a partition of the image pixels into regions, and of a polynomial surface inside each region. Then we describe the iterative process for obtaining a partition of the image into regions, with a polynomial surface model for reconstructing the depth inside each region, where the optimality criterion is the overall codelength for encoding (describing) the partition and the polynomial models for all regions, subject to a given allowed distortion over each region.

Given the disparity map image G we define a partition $P=\{\Omega_{\ell };\ell =1,\ldots ,L\}$ of the image support, $\Omega^{0}$, into *L* disjoint regions $\Omega_{\ell };\ell =1,\ldots ,L$, such that ${⋃}_{\ell =1}^{L}\Omega_{\ell }=\Omega^{0}$.

The minimum description length criterion consists of the cost L(P) of transmitting a segmentation $P_{n}$, evaluated by the implementable codelength obtained by context based coding of the segmentation [5], and of the cost $L(\Theta_{n})$ of encoding the parameters of all polynomial models. The precise cost of encoding any segment of the contour can be extracted during the coding process, and we will denote L(Γ) the codelength of encoding the contour segment Γ. We denote Γ(Ω) the outer contour of a connected region Ω.

For any given distortion *D* one needs to solve the optimization problem

(2) $$\begin{array}{c} \min_{P,\{\theta_{\ell }\}}\mathrm{RATE}=L\left(P\right)+\sum_{\Omega_{\ell }\in P}L\left(\theta_{\ell }\right)\\ \;\;\;\mathrm{subject}\;\mathrm{to}\;\;\;MSE=\frac{1}{n_{r}n_{c}}\sum_{\Omega_{\ell }\in P}\sum_{\left(i,j\right)\in \Omega_{\ell }}{\left(G\left(i,j\right)-S_{\theta_{\ell }}\left(i,j,\theta_{\ell }\right)\right)}^{2}\leq D. \end{array}$$

### 2.2. Algorithm for Hierarchical Segmentation based on Persistency of Contours of the Segmentations Generated by Iterative Piece-Wise Polynomial Modeling

The two components of the model are the following: (a) the segmentation and (b) the set of polynomial models, one for each region of the segmentation. Estimating the model that gives directly the minimum solution to the optimization problem (2) for a given *D* is approached by finding first a set of good “optimal” segmentations, and then checking what is the distortion corresponding to each segmentation, building thus a RD plot of solutions of (2).

The segmentation problem is sometimes seen as the estimation of a latent variable, defined for each pixel, and we introduced the label image notation X for this latent variable.

A simple attempt to finding a good model (including the segmentation X and the polynomial models) will be in the spirit of *K*-means iterative algorithm, rephrased as a *K*-models algorithm: fix a desired number of regions $N_{reg}$ in the segmentation and initialize a partition of the image into $N_{reg}$ regions. In a first stage, fit the best polynomial model over each region and in the second stage re-partition the image into $N_{reg}$ regions, so that each pixel (i,j) is associated with the model that gives the smallest reconstruction error of G(i,j). A true *K*-means or *K*-models would iterate the two stages until convergence, if that ever occur. However we are taking a different route: we operate with a large $N_{reg}$, in a very “over-segmented” regime, with $N_{reg}$ larger several times than the final intended maximum number of regions, and we are not interested in iterating until stabilization of the $N_{reg}$ regions. Instead we are interested in exploring as much variability in the region boundaries. Since the re-estimation will make the region to change their boundaries, we track during the process for each contour element, say H(i,j), the number of times in which it was part of regions boundaries during the process. We call counts(H(i,j)) the persistency degree of the contour element, and we are building our segmentations by considering progressively the contour elements in the decreasing order of their degree of persistency.

There are a few problems with the simple *K*-models approach, and we discuss them and introduce at the same time our algorithmic steps that are correcting the problems.

**Figure 2:**
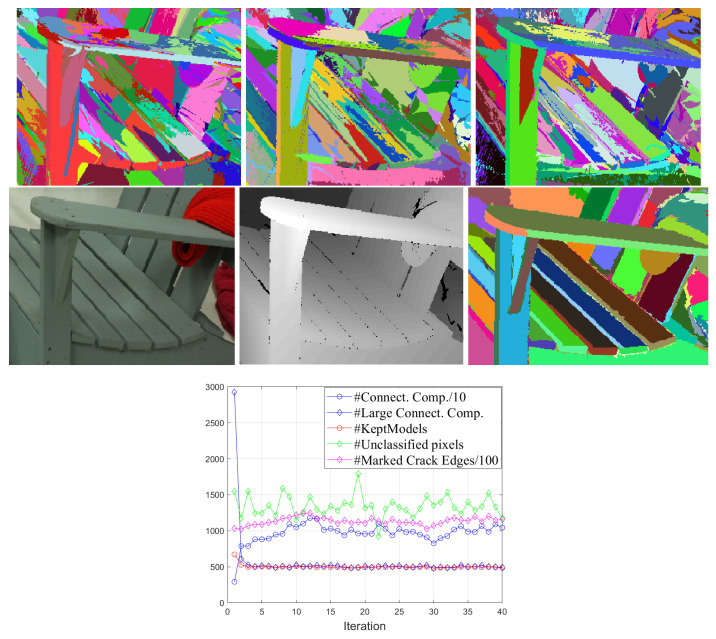
(Top row) Consecutive segmentations in the iterative re-estimation algorithm: *Panels 1 to 3:* First three consecutive segmentations in the iterative re-estimation algorithm over the disparity data; (Middle row) *Panel 1:* Detail of the RGB image; *Panel 2:* Detail of disparity image; *Panel 3:* The segmentation provided by the Algorithm 2, when including 234 regions for the entire image; The (200×300) pixels zoomed region is from Adirondack image of Middleburry dataset. (Bottom row) Plots with evolution over iterations of the variables in Algorithm 1.

We introduce several regularization options to this algorithm, resulting in the Algorithm 1. Even with the introduced change we notice that the iterative re-estimation has a high variability of the region contours decided at consecutive iterations. In Figure 2, we show on middle row on panels 1 and 2 one detail of the image Adirondack. The panels 1 to 3 in the top row show consecutive segmentations obtained during re-estimation. Since there are very many models initially (one for each (11×11) patch), on the long board which is the arm-rest of the chair there are several patches, with similar almost planar models, which are competing with each other during the re-estimation, and one sees the high variability of the contours of these models within the arm-rest in panels 1 to 3. However, the outline of the arm-rest remains as a clear part of region boundaries in all iterations. The main feature of our algorithm is to let many fitting polynomial surfaces to compete during the re-estimation iterations, resulting in many contour pixels that are changing from one iteration to the other, but also resulting in contour elements that remain “persistent” from one iteration to the next. We are keeping track of the persistency of all contour elements in the image, and after a number of iteration ($n_{iter}=40$ in all experiments) we check the persistency of each contour element and we use the most persistent elements for obtain contours that are true outlines of distinct objects or object parts. Just to show the final result of both Algorithms 1 and 2, we show in Figure 2, middle row, panel 3, that after selecting carefully the contour elements in Algorithm 2, using a rate-distortion marking of the regions, we are obtaining a segmentation very relevant for the object parts (the presented segmentation is obtained in Algorithm 2 after including persistent contours resulting in 243 regions in the segmentation image). In there, one can see that the collected persistent contours were successful in providing meaningful image features, resulting in a convincing segmentation.
**Algorithm 1** Hierarchical segmentations based on persistency of contours generated by iterative piece-wise polynomial modeling*Input:* The input disparity map G.**Stage 1.***Find persistent contours in the image G: Iterate finding the best fitting models for the current image partition, and then finding the best image partition for the current set of polynomial models. At each iteration mark the boundaries of the partition’s regions and add the binary edge matrix to the overall contour persistency matrix*; 1.0 Initialize the partition $P_{0}$ as being formed of $\left\lceil \frac{n_{r}}{L_{s}}\right\rceil \times \left\lceil \frac{n_{c}}{L_{s}}\right\rceil$ disjoint square regions $\left(L_{s}\times L_{s}\right)$. The corresponding label image is denoted $X_{0}$. The overall contour persistency matrix is set to $U=0\in R^{n_{r}\times 2n_{c}}$; 1.1 For $n=1,\ldots ,n_{iter}$*// Iterate a re-estimation algorithm $n_{iter}$ times*      1.1.1 *// Re-estimation iteration for finding a new set of models $\Theta^{\prime }=\left\{\theta_{\ell }|,\ell =1,\ldots ,n_{cc}^{\prime }\right\}$ and their number $n_{cc}^{\prime }$*       1.1.1.1 Decompose the image $X_{n-1}$ into connected components, denote $n_{cc}$ their number, and denote $P_{n-1}$ the partition into the regions $\Omega_{1},\ldots ,\Omega_{n_{cc}}$ so that $\Omega_{\ell }=\left\{\left(i,j\right)|X_{n-1}\left(i,j\right)=\ell \right\}$.       1.1.1.2 For each region $\Omega_{\ell }\in P_{n-1}$              1.1.1.2.1 If the cardinality of $|\Omega_{\ell }|$ is larger than $N_{S}$, estimate the parameters $\theta_{\ell }$ of the polynomial surface model by minimizing $\sum_{\left(i,j\right)\in \Omega_{\ell }}{\left(G\left(i,j\right)-P_{\theta_{\ell }}\left(i,j\right)\right)}^{2}$. Otherwise set the model $\theta_{\ell }$ to empty set.       1.1.1.3 Denote $n_{cc}^{0}$ the number of non-empty models estimated in previous step. Process the set of parameter vectors $\left\{\theta_{\ell }|,\ell =1,\ldots ,n_{cc}\right\}$ to select a subset$\Theta^{\prime }=\left\{\theta_{\ell }^{\prime }|,\ell =1,\ldots ,n_{cc}^{\prime }\right\}$ of $n_{cc}^{\prime }\leq N_{cc}$ models- If $n_{cc}^{0}>N_{cc}$, then group similar models together to obtain $n_{cc}^{\prime }\leq N_{cc}$ models.     1.1.2 *// Re-estimation iteration for finding a new partition $P_{n}$*      1.1.2.1 Initialization for the new $P_{n}$: number of regions $r_{n}=0$; reconstruction image $R_{n}=0$ and labels image $X_{n}=0$, with 0 the all zeros $\left(n_{r}\times n_{c}\right)$ matrix.      1.1.2.2 *// Use the competition of the models of $\Theta^{\prime }$ for defining the new partition*    For $\ell =1,\ldots ,n_{cc}^{\prime }$              1.1.2.2.1 Consider the model $\theta_{\ell }^{\prime }$              1.1.2.2.2 Initialize the winning binary image $B=0\in {\left\{0,1\right\}}^{n_{r}\times n_{c}}$, and then go over all pixels, for all $\left(i,j\right)\in \Omega^{0}$ and set B(i,j)=1 if $|P_{\theta_{\ell }}\left(i,j\right)-G\left(i,j\right)|<|R_{n}\left(i,j\right)-G\left(i,j\right)|$. Find all connected components of B and denote $\Omega^{*}$ the largest of them              1.1.2.2.3 If the cardinality of $\Omega^{*}$ is larger than a given size, $N_{S}$, then a new region is declared, r←r+1 and $\Omega_{r}=\Omega^{*}$              1.1.2.2.4 Include the new region $\Omega_{r}$ in the partition $P_{n}$, by updating the label image $X_{n}\left(i,j\right)=r$ and the corresponding reconstruction $R_{n}\left(i,j\right)=S_{\ell }\left(i,j\right)$, for each $\left(i,j\right)\in \Omega_{r}.$      1.1.2.3 Construct the contour image $C_{n}$ for $X_{n}$ and add it to the overall UCM matrix $U\leftarrow U+C_{n}$.**Stage 2.***Construct a hierarchical segmentation from the persistency contours matrix $U^{\prime }$, filtering out small regions*   2.1 For $i_{p}=n_{iter},n_{iter}-1,\ldots ,n_{min}$ (Iterate the persistency level from highest to smallest)      2.1.1 Construct a current contours image, C having C(i,j)=1 if $U\left(i,j\right)\geq i_{p}$.     2.1.2 Find the labels image X corresponding to C, and if $i_{p}=n_{iter}$ set $X_{i_{p}}=X$ and continue to $i_{p}=n_{iter}-1$     2.1.3 Find all connected components of the labels image X     2.1.4 Initialize $X_{i_{p}}=X_{i_{p}+1}$ (the labels of the previous partition)     2.1.5 For all connected components of the labels image X larger than $N_{2}$        2.1.5.1 If the connected component $\Omega_{\ell }$ has holes, fill each hole that is smaller than $N_{H}$ pixels and then copy the filled $\Omega_{\ell }$ to $X_{i_{p}}$ as a new region     2.1.5 Construct the contour map matrix $C_{i_{p}}$ corresponding to label image $X_{i_{p}}$ larger than $N_{S}$ and update the UCM matrix, $U^{\prime }\leftarrow U^{\prime }+C_{i_{p}}$   2.1 Rename the sequence $X_{n_{iter}},\ldots ,X_{n_{min}}$ as $X_{1},\ldots ,X_{N}$*Output:* The ultrametric contour map matrix $U^{\prime }$, and the sequence of segmentations $X_{1},\ldots ,X_{N}$, dubbed Hierarchical segmentations A.
**Algorithm 2** Hierarchical partition based on (description length - distortion) optimization*Input:* The sequence of segmentations $X_{1},\ldots ,X_{N}$ from Algorithm 1.**Stage 1.***Extract a catalog O of large regions (possible objects) from $X_{1},\ldots ,X_{N}$*      Each entry $O_{p}$ in the catalog corresponds to a large connected component region, $\Omega_{p}$, and is stored as a set of pixels $O_{p}^{S}=\Omega_{p}$   1.1 For r=1,…,N (Iterate from coarsest segmentation $X_{1}$ to finest segmentation $X_{N}$)     1.1.1 Find $\Omega_{1},\ldots ,\Omega_{m}$, all connected components of the labels image $X_{r}$     1.1.2 For q=1,…,m (go over $\Omega_{1},\ldots ,\Omega_{m}$)        1.1.2.1 If the size of $\Omega_{q}$ is smaller than 0.95 of the size of the parent region in $X_{r-1}$, but the cardinality $|\Omega_{q}|$ is larger than $N_{S}$, then the connected component is included in the catalog as a new region: p←p+1 and stored as $O_{p}^{S}=\Omega_{p}$.        1.1.2.2 Estimate the polynomial model parameters $\theta_{p}$.**Stage 2.***Construct a new sequence of segmentations $X_{1}^{\prime },\ldots ,X_{N}^{\prime }$ based on the (description length-distortion) optimization*    2.1 Initialize the current reconstruction image R=0 and the current label image $X_{0}^{\prime }=0$   2.2 For n=1,…,N (Add to $X_{n-1}^{\prime }$ a new region to form $X_{n}^{\prime }$)     2.2.1 For p=1,…,P (for all large regions from the catalog O that were not yet chosen)        2.2.1.1 Evaluate the candidate region $O_{p}$: find all regions $\Omega_{1},\ldots ,\Omega_{m}$ from $X_{n-1}^{\prime }$ overlapped (partially) by $O_{p}$.        2.2.1.2 For q=1,…,m (go over $\Omega_{1},\ldots ,\Omega_{m}$)          2.2.1.2.1 If the MSE of the current reconstruction R=0 over the $\Omega_{q}$ is better than the MSE over $\Omega_{q}$ of the surface generated by $\theta_{p}$, then carve out the set $\Omega_{q}$ from the candidate object: $O_{p}^{S}\leftarrow O_{p}^{S}\setminus \Omega_{q}$        2.2.1.3 If the remaining size of the region $O_{p}$ is larger than $N_{S}$, denote *r* the largest label of $X_{n}^{\prime }$ and set in $X_{n}^{\prime }$ the pixels form $O_{p}^{S}$ as a new region with label r+1.        2.2.1.4 Fit a new polynomial model $\theta_{p}$ over $O_{p}^{S}$, and find ΔMSE, the improvement in the MSE over $O_{p}^{S}$ of the new polynomial surface, compared to the current reconstruction R        2.2.1.5 Find the description codelength for specifying the better reconstruction, i.e., the description length of the polynomial $L(\theta_{p})$ and the description length L(Γ) of the additional contour for specifying $O_{p}^{S}$. Construct the ratio $\lambda_{p}=\frac{\Delta MSE}{L\left(\theta_{p}\right)+L\left(\Gamma \right)}$.     2.2.2 Pick from all candidate regions the one with the highest $\lambda_{p}$ and call the winning candidate index $p^{*}$.   2.3 If $\lambda_{c^{*}}$ is smaller than a threshold $\lambda_{0}$, stop adding regions and exit, else add the new region, by modifying $R_{n}$ and $X_{n}$ accounting for $O_{p^{*}}^{S}$.*Output:* The sequence of segmentations $X_{1}^{\prime },\ldots ,X_{N}^{\prime }$ dubbed Hierarchical segmentation B.


Now we present the main particularities of running Algorithm 1 followed by Algorithm 2, as cures to the *K*-means clustering. First, we do not know a priori a suitable number of regions, corresponding to a given distortion level *D*. For that reason we are letting the number of regions $N_{reg}=n_{cc}$ to change during the re-estimation iterations, and $N_{reg}$ will be decided implicitly by the selection decisions at each step. We initialize the algorithm with square partitions for simplicity, with the square side 11 pixels. This is similar to the initialization of the segmentation algorithms based on super-pixels. The initial $N_{reg}$ is in the order of thousands, resulting in a heavy over-segmentation of the image.

A major problem of the partition re-estimation is that when distributing each pixel (i,j) to the model that achieves the smallest reconstruction error of G(i,j), there might be very many good models that represent well the other pixels within a neighborhood of (i,j), and then in the neighborhood of (i,j) there may be many different labels of winning models. A certain model might result in many winning patches distributed over the image, with each patch having many holes due to the many similar competitors.

To tackle this problem we adopt several changes to the simple *K*-models structure of the algorithm. We enforce that during the *n*th re-estimation of the partition, a given model has associated only one connected component (the largest one) out of all possible connected components where the model was winning over the best current reconstruction. We go over the models in such an order that first we treat the models having a smaller winning patch, and we sequentially mark the winning patches in a label image $X_{n}$, overwriting the labels created by earlier patches. At the end of this marking process the label image $X_{n}$ will remain with the labels of the models having large winning patches. This process is described in Algorithm 1 at the Step 1.1.2.2 *Use the competition of the models of $\Theta^{\prime }$ for defining the new partition*. The label image $X_{n}$ can remain with many undecided pixels, since we restricted the marking of the winning patches to be only (large) connected components. All pixels with label 0 will be considered again in the decomposition of $X_{n}$ into connected components, at next run of the Step 1.1.1.1., and hence the number of models considered again in Step 1.1. may grow again larger than $N_{cc}$.

The number of models $n_{cc}^{0}$ that are re-estimated based on the new partition might be too large, exceeding our desired level $N_{cc}$. We use a very simple reduction of their number, by grouping together the “similar” models in the following way: we quantize each model with decreasing precision, by quantizing $Q\left(\theta_{\ell ,r}\right)=\left\lfloor \theta_{\ell ,r}2^{n_{b}}\right\rceil$ for $n_{b}=10,9,\ldots ,-10$ and for each $n_{b}$ we check how many quantized models are distinct in the sequence of parameter vectors $Q\left(\theta_{1}\right),\ldots ,Q\left(\theta_{n_{cc}}\right)$, picking the $n_{b}$ as the first number for which $n_{cc}$ remains below $N_{cc}$. This is the process described at the Step 1.1.1.3.

At each iteration of the re-estimation process we pick the contours elements set to 1 in $C_{X_{n}}$ and increment the contour matrix U at the corresponding locations. The contour matrix is a $\left(n_{r}\times 2n_{c}\right)$ matrix, where the first half block $U(1:n_{r},1:n_{c})$ specifies that the labels at X(i−1,j) and X(i,j−1) are different (horizontal edge) and the second half $U(1:n_{r},\left(n_{c}+1\right):2n_{c})$ specifies that the labels at X(i,j−1) and X(i,j) are different (vertical edge).

When the re-estimation iterations of Stage 1 are finished, we pass to Stage 2, to analyze the persistency levels marked in the matrix U, with the maximum possible value of $n_{iter}$. At each persistency level $i_{p}$ we create the contour matrix and then find the associated label matrix $X_{i_{p}}$. We want to avoid too small regions in $X_{i_{p}}$, and for that we decompose the image into connected components, fill for each of them the holes that are smaller than a fixed $N_{H}$ (we have used $N_{H}=50$) and use the filled connected components for a new label in $X_{i_{p}}$. The detailed description of Algorithm 1 is presented in the panel of the Algorithm 1.

To illustrate the re-estimation process, we show in Figure 2 bottom panel the evolution of some of the meaningful variables in Algorithm 1, Stage1: the number of connected components found in Step 1.1.1.1 $n_{cc}$ is marked *#Connect. Comp.*; The number of models estimated at the large connected components, $n_{cc}^{0}$ is marked *#Large Connect. Comp.*; The number $n_{cc}^{\prime }$ of models forced to be smaller than $N_{cc}$ is marked *#Kept Models*; The number of pixels remaining unclassified (unlabeled) after Step 1.1.2.2 is marked *# Unclassified pixels*; finally, the number of contour elements (crack edges) in H and V that are set to one at Step 1.1.2.3 is marked *#Marked Crack Edges*. It is seen that the variables in the re-estimation algorithm are changing at each iteration, inducing variability in the segmentations obtained at each iteration, which is our main goal in the iteration process.

### 2.3. Algorithm for Hierarchical Segmentation based on (Description Length-Distortion) Optimization

The Algorithm 2 starts at Stage 1 with creating a library of large regions (called now “objects” for simplicity, although no “object” meaning is claimed), by inspecting the sequence of segmentations obtained by Algorithm 1. The objects are allowed to overlap (but more than 95% is not allowed). An object is described by its set of pixel locations. For each such object the best model for reconstructing G is found and stored for later use.

At Stage 2 there is a competition between the objects, for being included as new labels in the label matrix $X_{n}^{\prime }$. The benefits in terms of MSE for including each candidate object with new labels in $X_{n}^{\prime }$ are evaluated, and possibly some subsets of the candidate object are removed, if the current reconstruction at the subset is better than that provided by the object. The best fit over the (carved) object is computed and the improvement in distortion ΔMSE is evaluated.

In order to solve the rate-distortion problem (2) the typical way is to evaluate the slope of the RD curve $\lambda_{p}=\frac{\Delta MSE}{L\left(\theta_{p}\right)+L\left(\Gamma \right)}$ and to add the objects to the segmentation in the order of their slope. The cost of the polynomial models is explained in Section 3.4. We denote Γ the set of additional contour elements that will be set to 1 due to the setting of the new region label in $X_{n}^{\prime }$. The cost for encoding Γ is estimated as being proportional to the cardinality of the set Γ (with a proportionality factor 1.5 found to cover experimentally well the cost of coding contour elements by the algorithm CERV). The candidate region having the largest value of λ is selected and the Algorithm 2 proceeds to find a new region, exiting when no region with improvements of ΔMSE can be found.

## 3. Experimental Results

### 3.1. The Datasets

**Figure 3:**
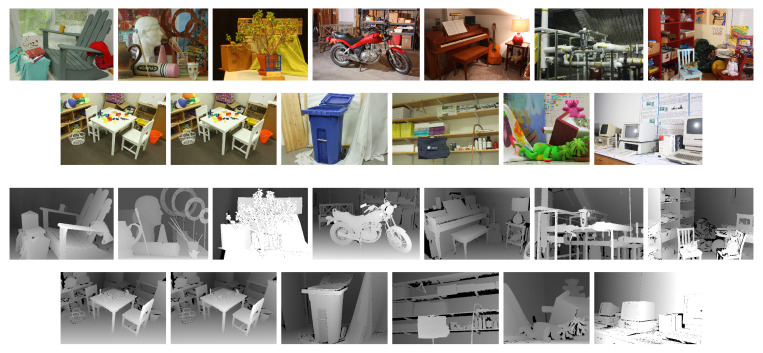
The RGB images and the corresponding disparity maps of the scenes from Middleburry dataset used in the experiments.

We have experimented with a dataset of high-resolution disparity images [36], where all the images were acquired from real scenes. In Figure 3 we show the scenes used. For the drafting of our algorithms and for setting the thresholds we have experimented over a set of synthetically generated images, but for space economy we do not present here results for the synthetic data. We emphasize that no threshold or algorithmic routines were tuned over the real data.

The dataset [36] was constructed for benchmarking of stereo matching algorithms, and it contains for each scene a left and a right color image, and also a left and a right disparity image. The disparity values can be assumed to be approximately inverse proportional to the depth values in the stereo setting, and then one could apply the algorithms to the modeling of the depth values (equivalent to inverse disparities), but we did not follow that route. Applying the polynomial surface models to depth images and to disparity images can produce rather different results. In [36] the goal was to produce high resolution *disparity* images, which were carefully evaluated and found to have approximately a quarter of a pixel precision when checking the stereo correspondence of the left and right color views. Hence we decided to apply the polynomial surface approximation directly to the disparity image data provided in [36], not to the inverse disparity data.

### 3.2. Obtaining the Sequences of Segmentations A and B

Our Algorithms 1 and 2 were run over the left disparity images of 13 scenes, on the quarter sized images, e.g., the image *Adirondack* has size (496,718). The left color images and left disparity map for the 13 scenes are shown in Figure 3. We have obtained for each scene two sequences of segmentations, A and B, using the Algorithms 1 and 2.

The number of segmentations produced by Algorithm 1 is maximum 38, since we have used $n_{iter}=40$, and we stopped the iterations of Step 2.1 at $n_{min}=3$, hence the maximum persistency level were 40. However, for some scenes the segmentations obtained at two consecutive values of $i_{p}$ where identical (especially at the very high $i_{p}$ values). The size threshold $N_{S}$ in both Algorithms 1 and 2 was set to 100.

The Algorithm 2 was producing a much larger number of segmentations, in the order of several hundreds, since we have used as the exit threshold condition in Step 2.3 $\lambda_{0}=0$, i.e., we exited when no large region had still associated a positive improvement gain λ.

### 3.3. Benchmarking the Sequences of Segmentations against References Extracted from the Color Images

The dataset also contains RGB images of the scenes, that we used only for finding color based contour maps or segmentations so that we can compare the segmentations obtained by our algorithms with other segmentation methods.

In order to find a reference contour map based on the RGB image we have used the software provided in a recent convolutional neural network (CNN) method [16] that performs edge detection, which was shown to achieve similar F-values as the human annotations over the BSD benchmarking database.

**Figure 4:**
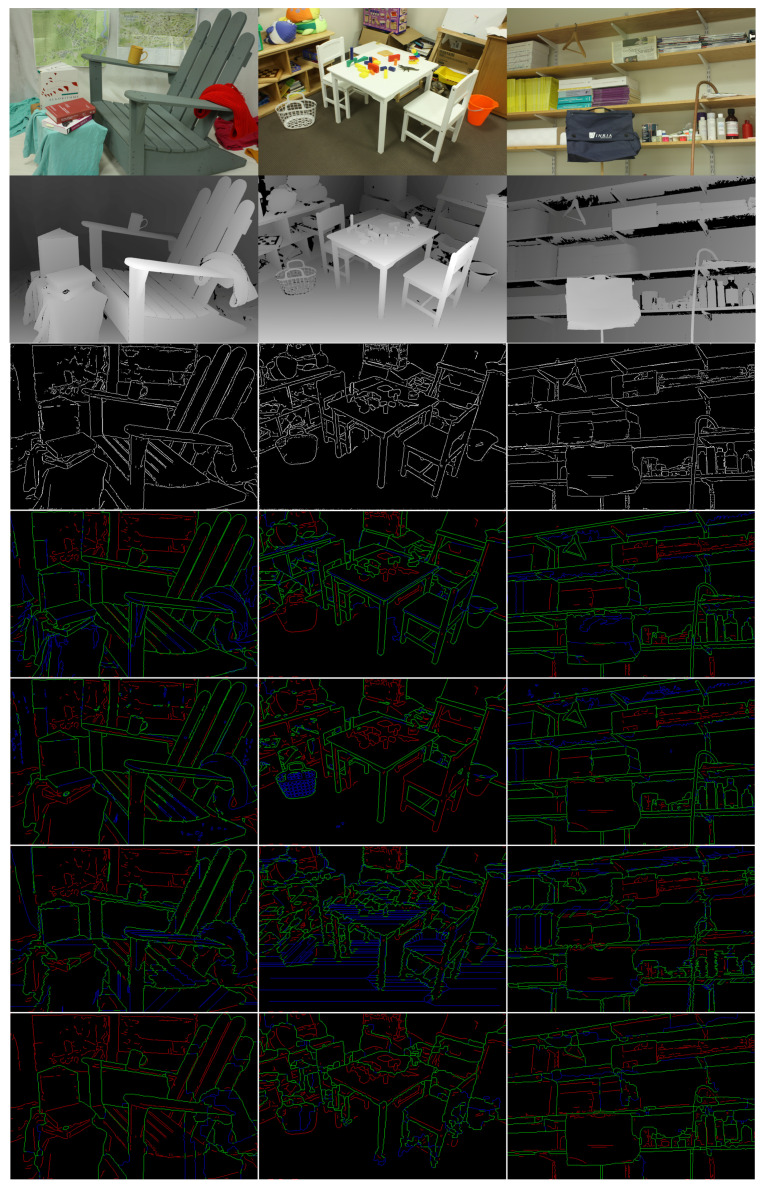
Matching the reference color edges (third row) by Algorithm 1 (fifth row), Algorithm 2 (fourth row), SSS algorithm [18] (sixth row) and by MCG algorithm [19] (last row): the green lines are true positives and blue lines are false positives (together they form the edges found by the algorithm on disparity image); the red lines are true negatives (edges existing in the reference color segmentation, but not found by the algorithm in the disparity image).

We have used the edge map produced for every RGB image of a scene by the software associated to [16], to obtain the edge map probability at every pixel, and we used additionally the non-maxima suppression from [14] for obtaining thin edges. We used a threshold for cutting the edge map probability for obtaining a map of binary edges, and by varying the threshold we obtained candidate contour maps of the image. By visually analyzing the contour maps at various thresholds, we have chosen for each scene one threshold, resulting in a computer generated “color reference edge map”, where the threshold was chosen by human. Since there are no public available annotations of the contours in Middleburry images, we chose to provide a substitute of the ground truth that we need for benchmarking by this reference edge map, generated with state of the art CNN based edge detection. We show in third row of Figure 4 the edge maps of the scenes, that we consider as reference color edge maps. They are used as a reference for benchmarking the performance of four algorithms: first: the segmentations generated by Algorithm 1, second, generated by Algorithm 2, (both obtained from *disparity data*), third, the segmentations obtained by the method Selective Search [18] dubbed here “SSS algorithm” using the *disparity data*, and fourth the segmentations obtained by the method Multiscale Combinatorial Grouping [19] dubbed here “MCG algorithm” using the *disparity data*, all against the reference color edge maps obtained from the RGB image.

**Figure 5:**
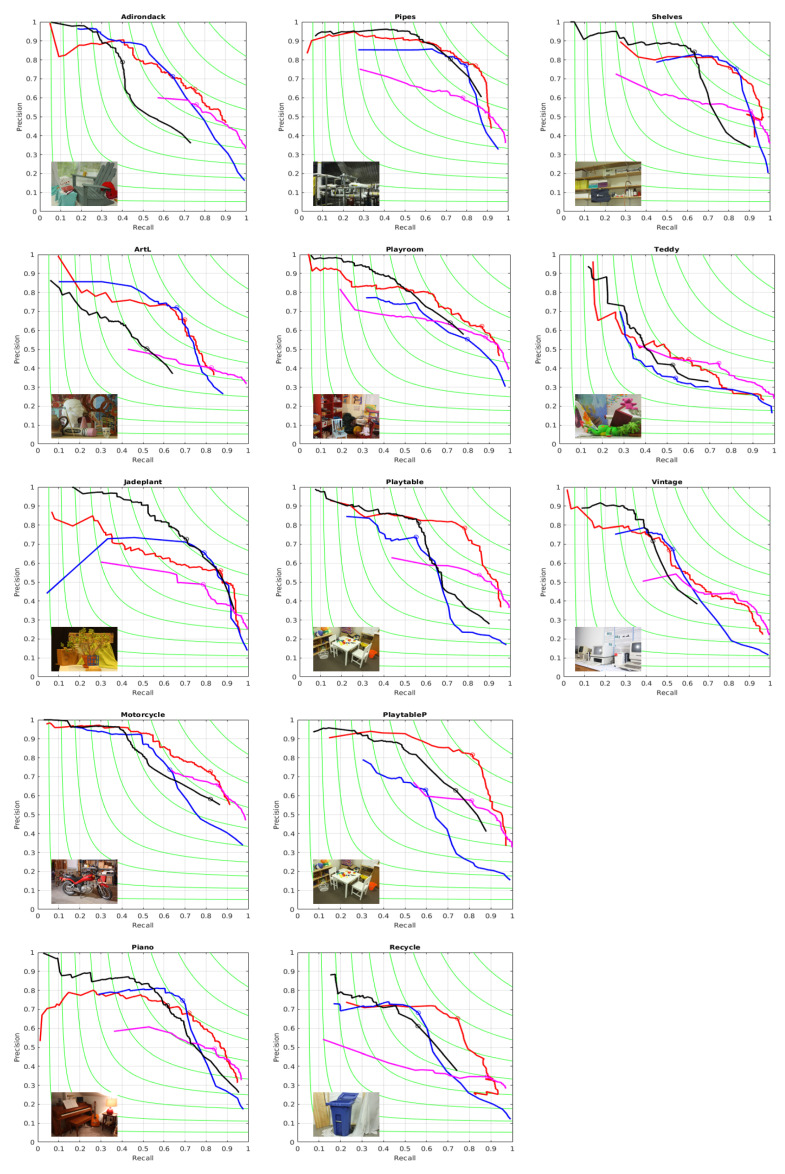
Boundary Precision-Recall Curves obtained with Algorithm 1, Algorithm 2, SSS algorithm [18] and MCG algorithm [19] for disparity images from Middlebury Dataset [36]. Algorithm 1: Blue, Algorithm 2: Red, SSS algorithm: Magenta, MCG algorithm: Black. F-value remains constant on each green curve.

For benchmarking we have used the established methodology for boundaries evaluations from [35] summarized next: one considers a binary edge map $B_{ref}$ as “ground truth”, which we take to be the reference color edge map mentioned above. Then we take say sequence A of segmentations, and go over each segmentation $X_{t}$, transform it to a binary boundary image $B_{t}$ (function provided in the software [35]) and then the contours in $B_{ref}$ and $B_{t}$ are aligned by a dynamic programming routine, resulting in the best alignment, and in two matching images, out of which one can obtain the true and false positive and the true and false negative, the precision $P_{t}$ and the recall $R_{t}$, and finally the *F* values $F_{t}=2P_{t}R_{t}/\left(P_{t}+R_{t}\right)$. For the best *F*-value for each image we also show the matching maps in Figure 4, where one can notice that the Algorithms 1 and 2 generate meaningful boundaries (the set of green and blue edges) and miss contour elements that are associated to color features, but not to disparity features. We present a complete description of the (precision-recall) performance in Figure 5 for all the images and all four algorithms and show by a circle the place with maximum *F* on each curve. In the Figure 5 and in the rest of the paper we refer to the method from [18] as SSS algorithm and to the method from [19] as MCG algorithm.

We also show in Table 1 the *F* values obtained for each algorithm over each image from the dataset. One can notice that Algorithm 2, based on the optimization using the gains λ defined in term of codelength, is the most often winner.

**Figure 6:**
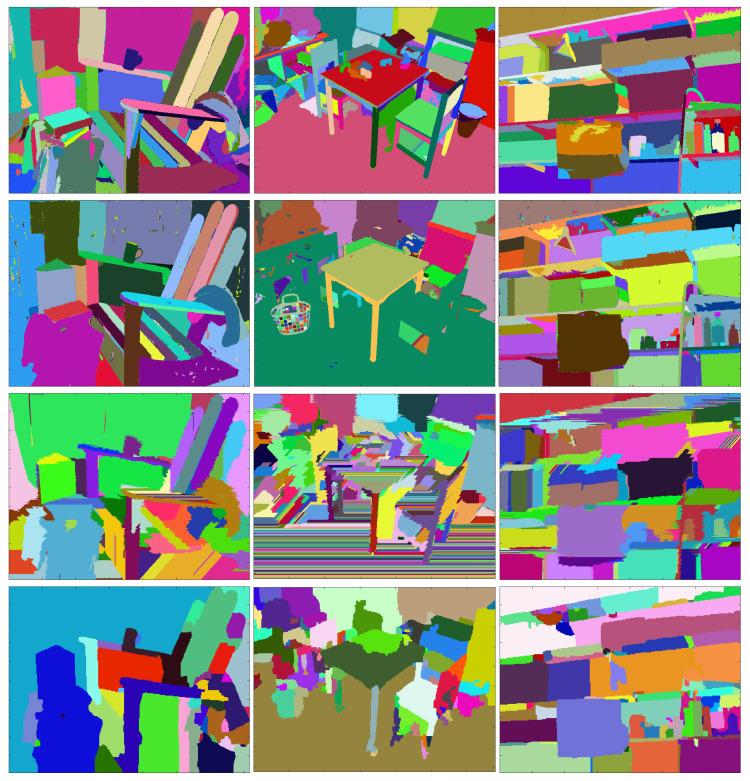
The segmentation corresponding to the best F-value obtained by Algorithm 2 (first row), Algorithm 1 (second row), by SSS algorithm [18] (third row) and by MCG algorithm [19] (last row).

Finally, we show for visual evaluation in Figure 6 the segmentations of the four compared methods, at the highest value of *F*. One can notice the good matching of the segmentation regions with the objects, or distinct parts of the objects in the scene.

### 3.4. Rate-Distortion Performance of the Segmentation Algorithm

We have used the encoding algorithm presented in Algorithm 3 for compressing G using the sequences obtained at the output of the Algorithm 1, Algorithm 2, SSS algorithm [18] and MCG algorithm [19]. The polynomial models have the parameter vector θ of length 6, corresponding to quadratic polynomial surfaces (1). For each obtained θ we quantize the parameters to 8 bits in the fractional part, and we encode the 6 quantized numbers by Golomb–Rice codes, determining the optimal parameter $k=2^{r}$ of the GR codes, where r∈{1,…,8} is also encoded (in three bits) and transmitted in the header of the bitstream. For each scalar coefficient θ we transmit in one bit the sign of the coefficient, in unary coding the quotient $q=\left\lfloor \frac{\left|\theta \right|}{2^{r}}\right\rfloor$ (i.e., *q* bits one followed by a 0 bit) and in *r* bits we transmit the binary representation of $\left(|\theta |-q2^{r}\right)$. The codelength for θ for each r∈{1,…,8} is evaluated and the optimal value of *r* is selected. The length of the bitstream for encoding the parameter vectors $\theta_{1},\ldots ,\theta_{L}$ is the model parameter cost $\sum_{\ell =1}^{L}L\left(\theta_{\ell }\right)$. Additionally, the cost L(X) of transmitting the segmentation X by using the CERV algorithm [5] needs to be added, to obtain the overall codelength L.
**Algorithm 3** Encoding G based on the segmentation X and polynomial models over each region*Input:* The input disparity map G. The segmentation X.   **Stage 1.** Encode the segmentation X by the CERV encoding algorithm from [5], resulting in the codelength L(X).     3.1 For ℓ=1,…,L (for each region $\Omega_{\ell }$ of the segmentation X)              1.1.1.2.1 Estimate the parameters $\theta_{\ell }$ of the polynomial surface model by minimizing $\sum_{\left(i,j\right)\in \Omega_{\ell }}{\left(G\left(i,j\right)-P_{\theta_{\ell }}\left(i,j\right)\right)}^{2}$.              1.1.1.2.1 Encode the parameters $\theta_{\ell }$ using a Golomb–Rice code, resulting in the codelength $L(\theta_{\ell })$.              1.1.1.2.1 Compute the sum of squares $SSE_{\ell }=\sum_{\left(i,j\right)\in \Omega_{\ell }}{\left(G\left(i,j\right)-P_{\theta_{\ell }}\left(i,j\right)\right)}^{2}$ of the reconstruction using the parameters $\theta_{\ell }$              1.1.1.2.1 Compute the mean square error $MSE=\frac{1}{n_{r}n_{c}}\sum_{\ell =1}^{L}SSE_{\ell }$ and the peak signal to noise ratio $PSNR=10\log_{10}\frac{Mg^{2}}{MSE}$ with $Mg=\max_{\left(i,j\right)\in \Omega^{0}}G\left(i,j\right)$.*Output:* The PSNR in dB and the rate $RATE=\frac{1}{n_{r}n_{c}}(L\left(X\right)+\sum_{\ell =1}^{L}L\left(\theta_{\ell }\right)$ in bits per pixel


We additionally consider the rate-distortion of other two lossy compression methods: first the wavelet based coder JPEG 2000, which uses a hierarchical wavelet decomposition and context based coding of the wavelet coefficients, in a heavily engineered reference software. Second we consider a simple method, dubbed “Model 0+UQ”, which performs first the uniform quantization (UQ) of the image G, with various quantization steps, and then transmits the quantized image using the CERV algorithm. For reconstruction, the scaling back by the quantization step is performed. Qualitatively, the quantized version of the image G looks like a geodesic map, being formed of constant regions enclosed by simple boundaries. This image can be encoded extremely efficiently by CERV, and hence “Model 0+UQ” has a very good RD curve, better than JPEG 2000 starting from a given rate on. However, at low bitrates the “Model 0+UQ” is below the JPEG 2000, and this is the place where the Algorithms 1 and 2 are intended to be utilized, for conveying at a low bitrate simple efficient reconstructions of G, which additionally convey a good segmentation into objects or parts of objects. The “Model 0+UQ” and JPEG 2000 competing methods do not provide informative segmentations, so we can compare them to ours only on the compression performance.

**Figure 7:**
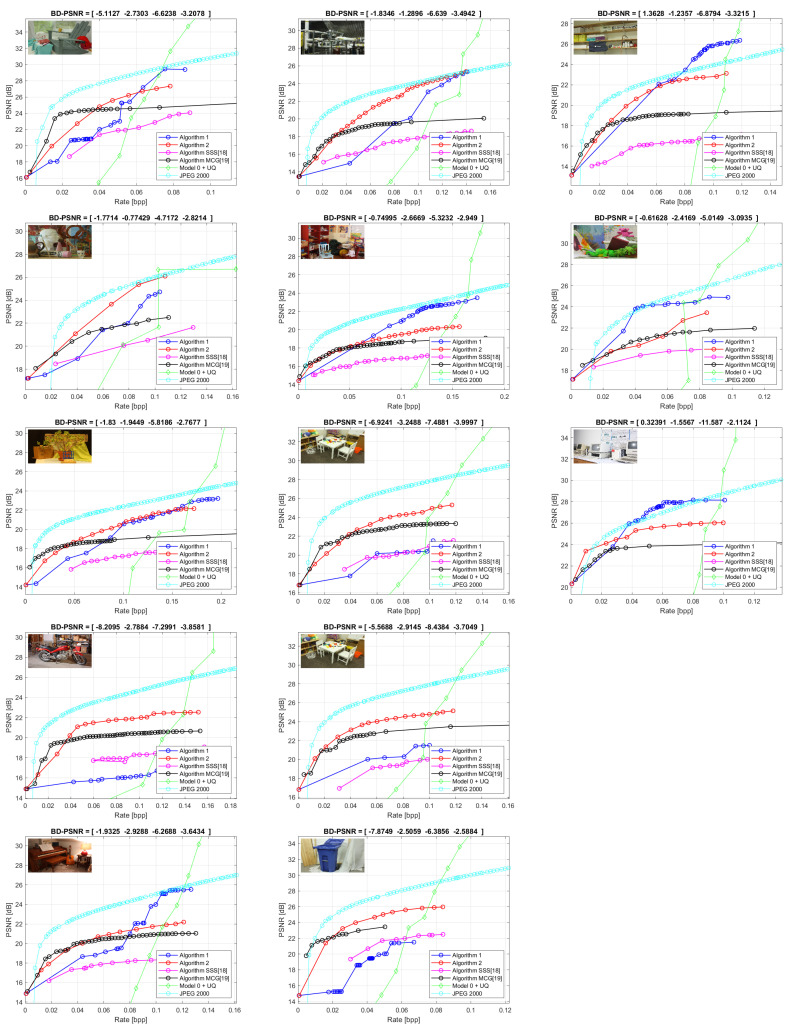
Rate-Distortion Curves obtained by segmentations generated by Algorithm 1, Algorithm 2, SSS algorithm [18], MCG algorithm [19], JPEG 2000 and Model 0+UQ.

We show in Figure 7 the Rate-Distortion plots for all the images and algorithms, in which we present the rate in normalized form, $L/(n_{r}n_{c})$, as bits per pixels. The performance of Algorithms 1 and 2 is better than “Model 0+UQ” at low bitrates, but still not as high as JPEG 2000. At the very low bitrate Algorithm 2 is systematically the best, but for some images Algorithm 1 succeeds to overpass significantly Algorithm 2 at higher bitrates. Finally, we show in Table 1 the Bjøntegaard distances (BD-PSNR) between the RD curves of each algorithm and the RD plot of JPEG algorithm. The minus sign for BD-PSNR means that the average of $\left(PSNR_{Alg}\left(R\right)-PSNR_{JPEG}\left(R\right)\right)$ is negative. The RD performance, for reconstructing the G, has to be seen in connection to the performance for boundary detection and object detection, which favors consistently Algorithm 2.

## 4. Conclusions

We have proposed algorithms that create segmentations from disparity images, useful for two goals: as segmentation of the scene, and also as partitions for a piece-wise polynomial model based lossy compression. These algorithms can be further combined in more complicated structures to cover the more advanced applications mentioned in the paper. The investigation in this paper revealed good properties of the created partitions for high resolution disparity images, encouraging further study of these models for the new types of immersive image modalities that may benefit from precise geometry modeling and compression.

## Figures and Tables

**Table 1:** Boundary-Recall and compression performances of hierarchical segmentation using four different segmentation algorithms (Algorithm 1, shortened as A1; Algorithm 2, shortened as A2; SSS algorithm [18]; and MCG algorithm [19])). The columns 2 to 5 present the optimal Precision-Recall pairs. The columns 6 to 9 present the optimal corresponding F-values. The columns 10 to 13 present the Bjøntegaard BD-PSNR values between the rate -distortion results when applying the encoding algorithm from Algorithm 3 to each of the four segmentation algorithms, compared against the rate-distortion of JPEG2000, considered as anchor. The bold results present the winning of the fours algorithm for each scene according to F-Value and according to Bjøntegaard BD-PSNR.

	Precision-Recall				F-Value				Bjøntegaard BD-PCNR (dB)			
Scene	A1	A2	SSS	MCG	A1	A2	SSS	MCG	A1	A2	SSS	MCG
Adirondack	0.71–0.64	0.65–0.75	0.56–0.76	0.79–0.40	0.68	**0.69**	0.65	0.53	−5.11	**−2.73**	−6.62	−3.21
ArtL	0.72–0.66	0.66–0.70	0.40–0.83	0.50–0.52	**0.69**	0.68	0.54	0.51	−1.77	**−0.77**	−4.72	−2.82
Jadeplant	0.66–0.79	0.56–0.87	0.49–0.79	0.73–0.71	0.72	0.68	0.60	**0.72**	**−1.83**	−1.94	−5.82	−2.77
Motorcycle	0.72–0.64	0.73–0.82	0.66–0.86	0.58-0.82	0.68	**0.77**	0.74	0.68	−8.21	**−2.79**	−7.30	−3.86
Piano	0.75–0.69	0.68–0.72	0.49–0.84	0.72–0.62	**0.72**	0.70	0.62	0.66	**−1.93**	−2.93	−6.27	−3.64
Pipes	0.77–0.80	0.77–0.84	0.60–0.78	0.80–0.72	0.78	**0.80**	0.68	0.76	−1.83	**−1.29**	−6.64	−3.49
Playroom	0.55–0.80	0.62–0.87	0.57-0.88	0.63–0.73	0.65	**0.72**	0.69	0.68	**−0.75**	−2.67	−5.32	−2.95
Playtable	0.74–0.55	0.78–0.78	0.54-0.85	0.81–0.57	0.63	**0.78**	0.66	0.67	−6.92	**−3.25**	−7.49	−4.00
PlaytableP	0.63–0.60	0.82–0.81	0.57–0.81	0.63–0.74	0.61	**0.82**	0.67	0.68	−5.57	**−2.91**	−8.44	−3.70
Recycle	0.68–0.56	0.65–0.74	0.35–0.88	0.65–0.53	0.62	**0.70**	0.50	0.58	−7.87	**−2.51**	−6.39	−2.59
Shelves	0.75–0.84	0.76–0.81	0.53–0.91	0.84–0.63	**0.79**	0.78	0.67	0.72	**1.36**	−1.24	−6.88	−3.32
Teddy	0.35–0.54	0.45–0.60	0.43–0.74	0.42–0.53	0.42	0.51	**0.54**	0.47	**−0.62**	−2.42	−5.01	−3.09
Vintage	0.67–0.53	0.66–0.52	0.44–0.82	0.72–0.43	**0.59**	0.58	0.57	0.54	**0.32**	−1.56	−11.59	−2.11
